# Taxonomic Compositions and Co-occurrence Relationships of Protists in Bulk Soil and Rhizosphere of Soybean Fields in Different Regions of China

**DOI:** 10.3389/fmicb.2021.738129

**Published:** 2021-09-17

**Authors:** Jun Zhang, Pengcheng Xing, Mengyu Niu, Gehong Wei, Peng Shi

**Affiliations:** State Key Laboratory of Crop Stress Biology in Arid Areas, Shaanxi Key Laboratory of Agricultural and Environmental Microbiology, College of Life Sciences, Northwest A&F University, Yangling, China

**Keywords:** protist community, bulk soil, rhizosphere, co-occurrence network, soybean fields

## Abstract

As the main consumers of bacteria and fungi in farmed soils, protists remain poorly understood. The aim of this study was to explore protist community assembly and ecological roles in soybean fields. Here, we investigated differences in protist communities using high-throughput sequencing and their inferred potential interactions with bacteria and fungi between the bulk soil and rhizosphere compartments of three soybean cultivars collected from six ecological regions in China. Distinct protist community structures characterized the bulk soil and rhizosphere of soybean plants. A significantly higher relative abundance of phagotrophs was observed in the rhizosphere (25.1%) than in the bulk soil (11.3%). Spatial location (*R*^2^ = 0.37–0.51) explained more of the variation in protist community structures of soybean fields than either the compartment (*R*^2^ = 0.08–0.09) or cultivar type (*R*^2^ = 0.02–0.03). The rhizosphere protist network (76 nodes and 414 edges) was smaller and less complex than the bulk soil network (147 nodes and 880 edges), indicating a smaller potential of niche overlap and interactions in the rhizosphere due to the increased resources in the rhizosphere. Furthermore, more inferred potential predator-prey interactions occur in the rhizosphere. We conclude that protists have a crucial ecological role to play as an integral part of microbial co-occurrence networks in soybean fields.

## Introduction

The plant rhizosphere, a “hotspot” environment that connects and differentiates root-associated and surrounding soil microbiomes, harbors an extraordinary number of microbes (Jiang et al., 2017) that can contribute to plant growth and plant health and provide ecological services (Chen et al., 2016). Recent advances in high-throughput sequencing (HTS) now let us investigate the diversity and function of these rhizosphere microbial communities (Mahoney et al., 2017), especially those composed of bacteria and fungi. Several studies have shown that the microbial communities that colonize the rhizosphere show distinct compositions and functions when compared with those of the surrounding bulk soil, largely due to specific soil physical structures, pH levels, and root exudates in the rhizosphere (Mendes et al., 2014; Edwards et al., 2015; Yan et al., 2016). The selective effect characteristic of rhizosphere-specific habitats leads to decreased microbial alpha diversity (Zhang et al., 2017, 2018a). Recent studies have indicated that rhizospheric microbial community structure is influenced by multiple factors, including spatial location, edaphic properties, plant genotypes, and root phenotypic traits (Peiffer et al., 2013; Edwards et al., 2015; Pérez-Jaramillo et al., 2017). In addition, the co-occurrence network architectures of rhizosphere microbial communities are less complex than those of surrounding bulk soil (Fan et al., 2017; Zhang et al., 2018c).

Most rhizospheric microbiome studies mainly focus on bacteria and fungi, leaving protists in the rhizosphere seldom investigated (Gao et al., 2019). Protists are a group of organisms with broad diversity that play multiple crucial roles in terrestrial ecosystems, functioning as primary producers, predators, food, and parasites. Free-living heterotrophic protists are considered the main consumers of bacteria, fungi and other microbes (Heger et al., 2018), while purely phototrophic and mixotrophic protists represent a functional soil carbon source (Jassey et al., 2015; Seppey et al., 2017), and protist parasites are widely distributed in soil and resistant cysts (Dupont et al., 2016). Protist activity can influence plant conditions by controlling the microbial loop (Bonkowski, 2004; Rossmann et al., 2020), altering hormonal balance in plants (Krome et al., 2010), stimulating plant growth-promoting rhizobacteria (Jousset, 2012; Asiloglu et al., 2020), and acting as plant-pathogenic species (Geisen et al., 2015). Furthermore, some protists affect other pathogens as antagonists or vectors (Gerbore et al., 2014; Xiong et al., 2020).

HTS based on 18S rRNA has been applied to investigate protist diversity in different ecosystems and habitats (Dupont et al., 2016; Mahé et al., 2017; Seppey et al., 2017). Importantly, HTS information may provide insight into functional ecology based on the genetic identification of protists and knowledge of their life histories (De Vargas et al., 2015; Weisse et al., 2016). In particular, the microbial relationships between protists and other microbes can be assessed using a co-occurrence network generated from the HTS data. For example, a recent study showed that protists linked diverse bacterial and fungal populations as a central hub to improve the health of different fertilizer-treated soils (Xiong et al., 2017). Despite these developments, we still lack necessary knowledge of how the taxonomic and functional composition of protists is structured and affected by environmental variables, such as climate, soil, and vegetation, and how protists are associated with their potential bacterial and fungal prey in the niches of bulk soil and rhizosphere compartments.

Soybean [*Glycine max* (L.) Merrill], as one of the most important crops in China, is distributed almost all over the country. Wang and Gai (2002) suggested that cultivating regions of soybean in China could be divided into six ecological regions. Our previous studies have explored rhizospheric bacterial and fungal communities of soybean in different ecological regions in China (Zhang et al., 2018a,b). However, we still lack essential knowledge about rhizospheric protist community assemblages of soybean in China.

To fill these pressing knowledge gaps, we carried out HTS based on 18S rDNA to investigate protist communities inhabiting the bulk soil and rhizosphere of three soybean (Glycine max) cultivars collected from six agricultural regions in China. Then, we linked the taxonomically assigned protist taxa to several functional groups of soil food webs. Furthermore, we also combined the HTS-obtained data on bacterial and fungal communities from 16S rDNA and ITS2 to explore the roles of protists in soil ecological networks. In this study, the following hypotheses were tested: (i) The taxonomic and functional compositions of protists in the rhizosphere differ from bulk soil; and (ii) Protist co-occurrence networks have features that differ between the two soil compartments, and protists play crucial roles in overall networks and link diverse bacterial and fungal populations.

## Materials and Methods

### Site Description and Sampling

Three soybean (Glycine max) cultivars (HH53, WH40, and ZH13) were planted in replicate plots (222 m^2^ each cultivar) in six fields under conventional cultivation. The soybean fields were located at different sites in China (Supplementary Table 1), including Heihe (HH), Chifeng (CF), Yan’an (YA), Wuhan (WH), Nanchang (NC), and Quanzhou (QZ). These locations are distributed across distinct ecological regions with greatly different edaphic and climatic factors. Soil samples were collected in each field at the stage of soybean flowering (between June and July 2015). There were 72 samples in total: 54 rhizosphere samples (6 locations × 3 cultivars × 3 repeats) and 18 bulk soil samples (6 locations × 3 repeats). Bulk soil samples were collected from each field site by mixing five soil cores obtained from the 0–20 cm soil layer after first removing any stones and plant debris. To obtain rhizospheric soils, we collected healthy soybean plants from five plots per field. Excess soil was removed from the roots by manual shaking, with approximately 1 mm of soil still attached to them, and this rhizospheric soil was collected by centrifugation after washing with a solution of sterile phosphate-buffered saline, following the method of Edwards et al. (2015). All samples were stored at –80°C before DNA extraction.

A subsample of bulk soil was air dried and then sieved to detect edaphic properties, namely, pH, C:N ratio, organic C, and total N; clay, silt, and sand contents; and available N, K, P, Ca, and Mg concentrations (Zhou and Zhang, 2008). Furthermore, we collected climatic data for the mean annual precipitation and temperature and (MAP and MAT), potential evapotranspiration (PE), relative humidity (RH), and aridity index (AI) from the China Meteorological Database.^1^ The above information is presented in Supplementary Table 1.

### Microbial Community Analysis

Total DNA was extracted from approximately 0.5 g of each sample by using the Fast DNA SPIN Kit (MP Biomedicals, United States) and following the instructions. The V9 region of the 18S rRNA gene was amplified with the eukaryote universal primer combination 1389F (TTGTACACACCGCCC)/1510R (CCTTCYGCAGGTTCACCTAC) (Amaralzettler et al., 2009); it contained a barcode sequence from each sample for Illumina MiSeq sequencing. All PCRs were carried out in a 25-μL volume containing 12.5 μL of 2 × Taq PCR MasterMix, 1 μM forward and reverse primers (5 μM), 3 μL BSA (2 ng/μL) and 30 ng template DNA, and ddH_2_O filled to 25 μl. Thermal cycling included initial denaturation at 95°C for 5 min, then 40 cycles of denaturation at 95°C for 45 s, annealing at 60°C for 50 s, and elongation at 72°C for 45 s, with a final elongation at 72°C for 10 min. The PCR products were pooled and cleaned using the Qiagen Gel Extraction Kit (Qiagen, Hilden, Germany) and sequenced with the Illumina MeSeq platform.

Raw sequences were processed to obtain high-quality tags (Caporaso et al., 2011). The USEARCH tool was used to remove chimeric sequences based on the UCHIME algorithm (Edgar et al., 2011). Sequences were clustered into operational taxonomic units (OTUs) of 97% similarity with UPARSE (Edgar, 2013). Then, these OTUs were blasted against the trimmed V9 region (V9_PR2) database (Guillou et al., 2012; De Vargas et al., 2015). Finally, any plant (Streptophyta), animal (Metazoa), fungal, and unclassified Opisthokonta OTUs were discarded. To better predict the potential ecological functions of different protist taxa, we further assigned them to six functional groups—phagotrophs, omnivores, parasites, phototrophs, and plant pathogens—according to a previous study (Xiong et al., 2017).

The V4 region of the bacterial 16S rRNA gene, using primer pairs 515F (GTGCCAGCMGCCGCGGTAA)/806R (GGACTACHVGGGTWTCTAAT), and the fungal internal transcribed spacer 2 (ITS 2) region, using primer pairs ITS3–2024F (GCATCGATGAAGAACGCAGC)/ITS4–2409R, were each amplified from the bulk soil and rhizospheric soil samples (Orgiazzi et al., 2012; Evans et al., 2014). A detailed description of the sequencing and processing procedure was performed according to the above instructions. The representative OTU sequences were aligned against the Silva database (Release 119) for bacteria and the UNITE database (Release 7.0) for fungi.

### Statistical Analysis

All analyses of our study were conducted in the R software platform v.3.4.5 (R Core Team, 2017) and QIIME v1.9.0 (Caporaso et al., 2010). The alpha diversity of each protist community was expressed by the number of observed OTUs (=richness) and its phylogenetic diversity (PD) index using a rarefied OTU table. Significant differences in alpha diversity between the bulk soil and rhizosphere were tested using the Wilcoxon test with *P* values adjusted using the Benjamini-Hochberg method (Benjamini and Hochberg, 1995). A differential OTU abundance analysis was performed by fitting a negative binomial generalized linear model (Zgadzaj et al., 2016) to identify the OTUs correlated with differences in protist communities between the bulk soil and rhizosphere by using the edgeR library (Robinson et al., 2010). The OTU counts of the bulk soil served as the control, and the *P* value cutoff was set to 0.05.

The “pcoa” function from the ape package (Paradis et al., 2004) and the “adonis” and “capscale” functions from the vegan package (Oksanen et al., 2013) were used to perform unconstrained principal-coordinate analysis (PCoA) and permutational multivariate analyses of variance (PERMANOVA) based on Bray-Curtis and weighted UniFrac distances, respectively. For these beta-diversity analyses, the OTU counts were normalized via the cumulative-sum scaling (CSS) method. Bray-Curtis and weighted UniFrac distances were calculated with the vegan (Oksanen et al., 2013) and phyloseq packages (McMurdie and Holmes, 2013). Canonical correspondence analysis (CCA) was performed to explore the edaphic and climatic factors influencing the protist communities of the bulk soil and rhizosphere. We relied on a forward selection procedure to select local edaphic and climatic factors, using the “ordiR2step” function from vegan to identify which were statistically significant (*P* < 0.05) to generate the CCA (Blanchet et al., 2008).

Additionally, we built protist co-occurrence networks and overall networks integrating the protist, bacterial and fungal community data to gain deeper insight into potential microbial interactions within the bulk soil and rhizosphere. The network construction was run with the Cytoscape plugin CoNet (Faust et al., 2012) using an ensemble approach that combined two measures of correlation (Spearman and Pearson) and dissimilarity (Kullback–Leibler and Bray-Curtis). Then, we requested the 1,000 top- and bottom-ranking links for each measure. Statistical significance was computed by obtaining the method and edge-specific permutation and bootstrap score distributions using 1,000 iterations for each distribution. The *P* values were merged using the method of Brown (1975) and corrected for multiple testing by the Benjamini-Hochberg procedure (Benjamini and Hochberg, 1995). Only links with an adjusted merged *P* value < 0.001 were filtered to obtain final networks. Further statistical analyses of these networks were conducted with the implemented tool NETWORKANALYZER (Assenov et al., 2007) and the igraph R package (Csárdi and Nepusz, 2006). We performed module-eigengene (the first principal component of modules) analysis to examine the correlations between main modules and environmental factors (Zhang et al., 2018c). The significant was tested by Mantel analysis.

The keystone species were identified based on *z*-score and *c*-score cutoffs. The network hubs (*z*-score > 2.5 and *c*-score > 0.6) were highly connected to the total network, the module hubs (*z*-score > 2.5 and *c*-score < 0.6) were highly connected within a network module, the connectors (*z*-score < 2.5 and *c*-score > 0.6) were responsible for linking among varied network modules, and the peripherals (*z*-score < 2.5 and *c*-score < 0.6) had few links with other nodes (Poudel et al., 2016). The OTUs belonging to connectors, network hubs and module hubs were identified as keystone species.

## Results

### Protist Community Structure and Diversity

**FIGURE 1:**
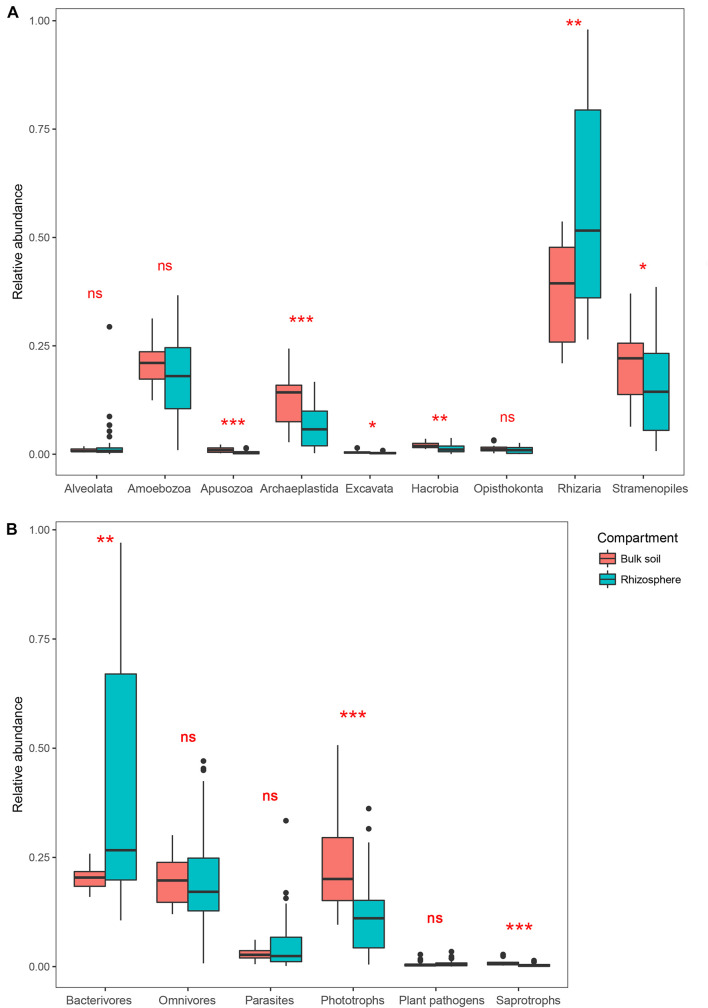
Boxplots showing mean (±SE) relative abundances of five main protist taxonomic groups **(A)** and protist functional groups **(B)** in the bulk soil (*n* = 18) and rhizosphere (*n* = 54) of soybean fields in China. Statistical differences were analyzed using the Wilcoxon tests: ***P < 0.001, **P < 0.01, *P < 0.05, and ns denotes a non-significant difference.

After removing the plant (Streptophyta), animal (Metazoa), fungal and unclassified Opisthokonta sequences, a total of 2,983 OTUs were identified as protists from our samples. Rhizaria, Stramenopiles, Amoebozoa, and Archaeplastida dominated the protist communities in both the bulk soil and rhizosphere (Supplementary Figure 1). Comparing the two compartments, the relative abundance of Rhizaria was significantly higher in the rhizosphere (Wilcoxon test, *P* < 0.05), whereas Apusozoa, Archaeplastida, Excavata, Hacrobia, and Stramenopiles were significantly less abundant in the rhizosphere (*P* < 0.05; Figure 1A). Protist OTUs were classified into six functional groups according to the method of Xiong et al. (2017). For these, the relative abundance of phagotrophs in the rhizosphere (25.1%) was more than twice that in the bulk soil (11.3%); however, the latter harbored at least twofold more phototrophs and saprotrophs than the rhizosphere in relative abundance (*P* < 0.05; Figure 1B).

**FIGURE 2:**
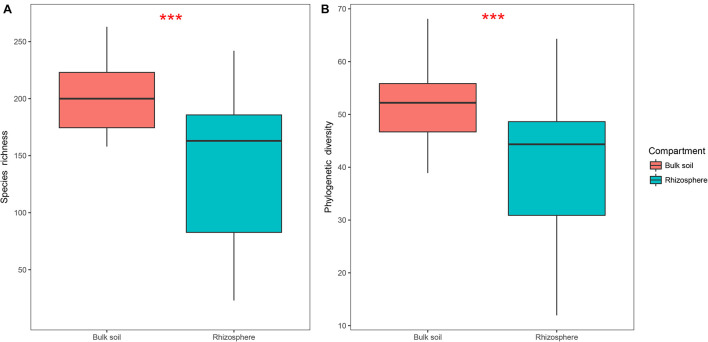
Boxplots showing species richness **(A)** and phylogenetic diversity **(B)** of the bulk soil (*n* = 18) and rhizosphere (*n* = 54) compartments in soybean fields of China. Statistical differences were analyzed using Wilcoxon tests: ***P < 0.001.

Protist alpha diversity expressed as richness and PD at the same depth of rarefied sequences (446 reads per sample) was significantly lower in the rhizosphere (Mean ± SE, 144 ± 60 for richness, 40 ± 13 for PD; Figure 2) than in the bulk soil (201 ± 32 for richness, 52 ± 7 for PD; Figure 2). A total of 39 OTUs were significantly enriched in the rhizosphere (Supplementary Figure 2A), which mainly consisted of Rhizaria (35.9%; Supplementary Figure 2B) and Amoebozoa (30.8%; Supplementary Figure 2B). Additionally, 1,025 OTUs were significantly depleted in the rhizosphere (Supplementary Figure 2A), which mainly consisted of Rhizaria (34.9%; Supplementary Figure 2C) and Alveolata (25.2%; Supplementary Figure 2C). Both phagotrophs (32.1%; Supplementary Figure 2D) and omnivores (39.3%; Supplementary Figure 2D) dominated the enriched OTUs, while phagotrophs (37.6%; Supplementary Figure 2E) and phototrophs (30.8%; Supplementary Figure 2E) dominated the depleted OTUs.

### Linkage Between Protist Community Structure and Environmental Factors

**FIGURE 3:**
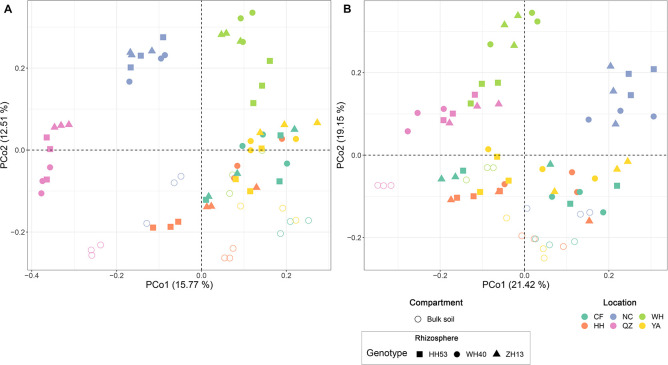
Principal coordinate analysis based on Bray-Curtis **(A)** and weighted UniFrac **(B)** distances the showing contribution of compartment, location, and cultivar on shaping the overall composition of root-associated protist communities of soybean fields in China.

Permutational multivariate analyses of variance analysis revealed that location represented the dominant source of variation (based on Bray-Curtis distances, *R*^2^ = 0.37, *P* < 0.001; on weighted UniFrac distances, *R*^2^ = 0.42, *P* < 0.001; Supplementary Table 2A), followed by compartment (Bray-Curtis, *R*^2^ = 0.08, *P* < 0.001; weighted UniFrac distances, *R*^2^ = 0.09, *P* < 0.001; Supplementary Table 2A). However, the cultivar grown did not significantly (*P* < 0.05) impact the protist communities of soybean fields (Bray-Curtis distances, *R*^2^ = 0.03, *P* = 0.08; weighted UniFrac distances, *R*^2^ = 0.02, *P* = 0.13; Supplementary Table 2B). We also used an unconstrained PCoA to confirm the PERMANOVA results (Figure 3). The first axis (PCo1) separated the communities mainly by location, especially those of NC, WH and QZ, while the second axis (PCo2) separated them mainly by compartment.

**FIGURE 4:**
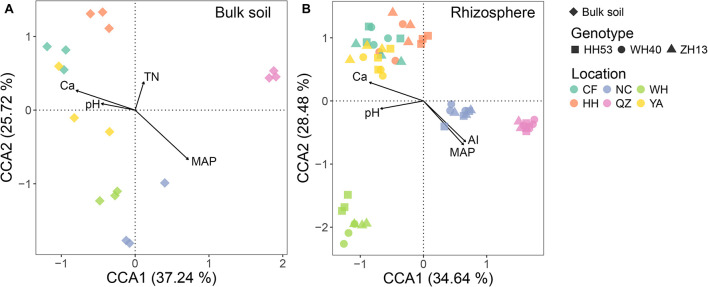
Canonical correspondence analysis (CCA) showing the correlations among environmental factors (edaphic and climatic factors) and protist communities from the bulk soil **(A)** and rhizosphere **(B)** of soybean fields in China. TN, total nitrogen; pH, soil pH; MAP, mean annual precipitation; AI, aridity index.

The influence of environmental factors (including edaphic and climatic factors) on protist community structure in the bulk soil and rhizosphere was qualified through CCA ordination. The combination of four environmental factors (pH, Ca, TN, and MAP for the bulk soil; pH, Ca, AI, and MAP for the rhizosphere; Figure 4) was significantly correlated with changes in the protist community for each compartment based on forward model selection (*P* < 0.05). Of all the environmental factors tested, MAP was the most important for driving the protist community in both the bulk soil and rhizosphere, since it had the longest arrow in the CCA. This result was also verified by the Mantel test, which revealed that MAP had the strongest correlation (*R* = 0.65, *P* < 0.001 in the bulk soil; *R* = 0.66, *P* < 0.001 in the rhizosphere; Supplementary Table 3) with shifts in the protist community compositions.

### Co-occurrence Networks of Protist Communities

Overall, the ecological networks were clearly unalike between the compartments. The rhizospheric network was smaller and less complex than the network of bulk soil. The bulk soil had a co-occurrence network consisting of 147 nodes and 880 edges (average degree of 11.97), which exceeded those of the rhizospheric network (76 nodes, 414 edges, average degree of 10.89). Furthermore, there was a larger proportion of positive correlations in the rhizosphere network (91.79%) compared with bulk soil (76.82%, Table 1).

**TABLE 1:** Topological properties of the empirical microbial co-occurrence networks in the bulk soil and rhizosphere of soybean fields in China and their associated random networks.

Parameters	Compartment	
	Bulk soil	Rhizosphere
**Empirical networks**		
Number of nodes	147	76
Number of edges	880	414
Number of positive correlations	676	380
Number of negative correlations	204	34
Average degree	11.97	10.89
Density	0.082	0.145
Average path length (APL)	2.706	2.431
Average clustering coefficient (ACC)	0.453	0.572
Modularity	0.47	0.41
**Random networks**		
APL (mean ± SE)	2.264 ± 0.004	2.030 ± 0.008
ACC (mean ± SE)	0.082 ± 0.005	0.146 ± 0.010
Modularity (mean ± SE)	0.235 ± 0.007	0.225 ± 0.009

**FIGURE 5:**
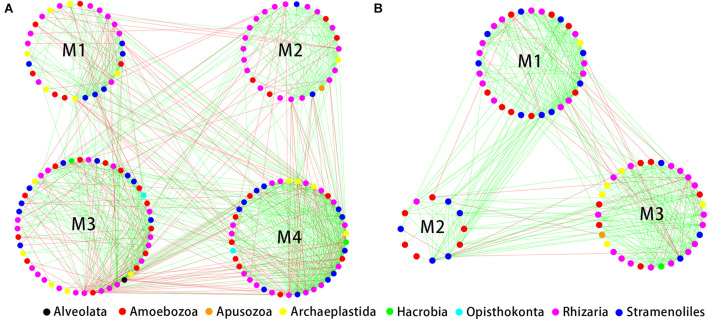
Correlation-based network analysis showing all potential interactions between protist operational taxonomic units (OTUs) in the bulk soil (**A**, *n* = 18) and rhizosphere (**B**, *n* = 54) of soybean fields in China. Node colors indicate different protist taxonomic groups. Lines connecting nodes (edges) represent positive (red) or negative (blue) co-occurrence relationships.

The indices of modularity, average path length (APL) and average clustering coefficient (ACC) of the above empirical networks were compared with those of random networks using *Z*-tests for the bulk soil and rhizosphere (Table 1). This analysis indicated non-random co-occurrence patterns in both the bulk soil and rhizosphere compartments (*P* values < 0.001). Greater APL and ACC values characterized each network when compared with corresponding random networks (Table 1), thus indicating that the empirical networks had “small world” properties. Modularity values were >0.4 (Table 1), indicating that each empirical network had a modular structure. We obtained four and three highly connected modules—each module with over six nodes—in the bulk soil and rhizosphere, respectively. Rhizaria dominated all four modules of the bulk soil network and two modules of the rhizospheric network in number, and Amoebozoa dominated the remaining module of the rhizospheric network (Figure 5).

Then, the investigated the correlations between modules and environmental factors by Mantel analysis. The correlations were mainly detected in specific modules. These modules (modules III and IV in the bulk soil, Supplementary Table 4A; module I in the rhizosphere, Supplementary Table 4B) were significantly related to a wide range of environmental factors. Other modules were rarely related to environmental factors.

### Co-occurrence Networks of Protists and Other Microorganisms

**FIGURE 6:**
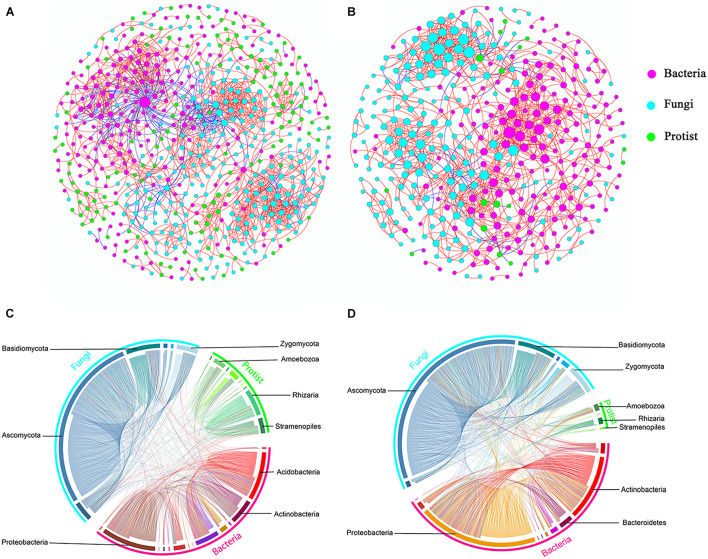
Co-occurrence networks constructed from total microbial communities in bulk soil (**A**, *n* = 18) and rhizosphere (**B**, *n* = 18), respectively, and the distributions of bacteria, fungi and protist **(C,D)**. Each node represents an OTU, the size of each node is proportional to the degree **(A,B)**. Red and blue lines connecting nodes (edges) represent positive and negative relationships, respectively **(A,B)**.

**FIGURE 7:**
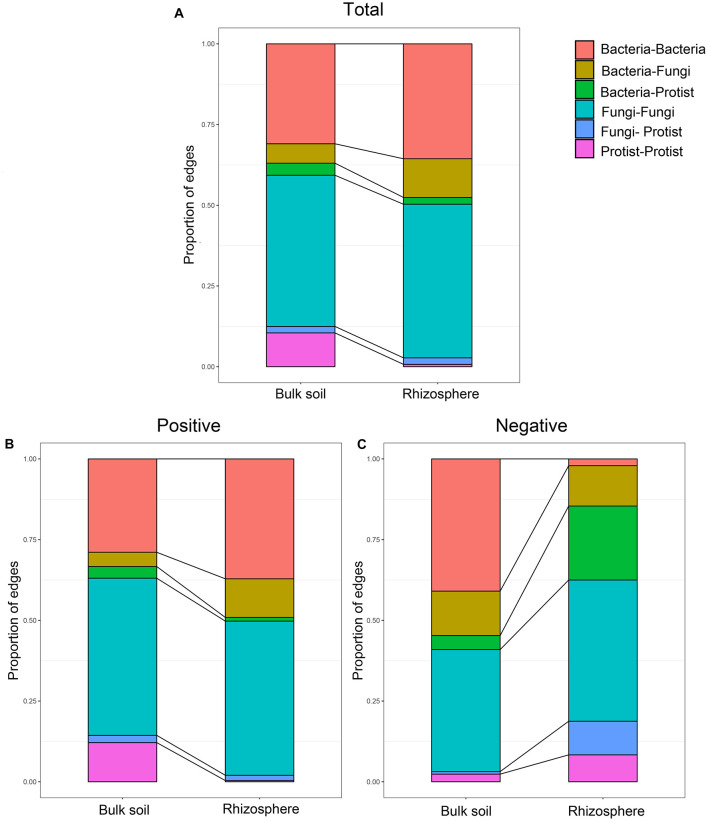
Relative abundance of each type of edge in the co-occurrence networks of the microbial communities in bulk soil and soybean rhizosphere, **(A)** for all edges, **(B)** for positive edges, and **(C)** for negative edges.

Further, we constructed overall networks based on protist, bacterial and fungal OTUs of the bulk soil and rhizosphere (Figure 6). The overall network in the bulk soil consisted of 601 nodes (225 bacterial OTUs, 237 fungal OTUs, and 139 protist OTUs) and 1,499 edges; the overall network in the rhizosphere consisted of 392 nodes (185 bacterial OTUs, 191 fungal OTUs, and 16 protist OTUs) and 1,093 edges (Supplementary Table 5). Similar to protist networks, the rhizosphere had a smaller and less complex integrated network compared with bulk soil. In bulk soil, the interactions mainly occurred in the same type of microorganisms, and the interaction between different types of microorganisms was less (Figure 6A). In the rhizosphere, protists mainly interact with bacteria and fungi (Figure 6B). The proportion of positive edges correlated with the protists decreased, while the proportion of negative edges correlated with the protists increased to varying degrees compared with the bulk soil (Figure 7). We analyzed the roles of different types of microbial nodes in the integrated networks to identify the keystone species. Protists were identified as keystone species in both the rhizosphere and the bulk soil. In the bulk soil, 2 protist OTUs were identified as module hubs, and 1 protist OTU was identified as a connector (Supplementary Figure 3A). In the rhizosphere, 1 protist OTU was identified as a module hub (Supplementary Figure 3B).

## Discussion

The symbiotic relationship between soybean and rhizobia is a supreme example of plant–microbe mutualism. Han et al. (2020) demonstrated an important role of the rhizospheric microbial community in shaping rhizobia-host relationships in soybean. Rhizospheric bacterial and fungal communities of soybean have been well investigated (Zhang et al., 2018a,b). Rhizospheric protists play various ecological roles in soil food webs and therefore display great functional versatility (Xiong et al., 2020). However, a comprehensive understanding of the diversity and ecological functioning of rhizospheric protists of soybean still eludes us. The development of new molecular markers and databases allows us to understand the rhizospheric protist community (Ceja-Navarro et al., 2021). On the other hand, exploring relationships between protists and other microorganisms was essential to comprehensively understand the ecological roles of the protist community in the soybean rhizosphere.

### Distinctive Protist Community Structures of Bulk Soil and Soybean Rhizosphere

Our results revealed that protist community structures were distinguishable between the bulk soil and soybean rhizospheric soil. Notably, the rhizosphere had a lower protist alpha diversity than that in bulk soil. A similar pattern has been well established elsewhere, in that the bacterial and fungal community structures in the rhizosphere differed from those in bulk soil in rice and wheat fields (Edwards et al., 2015; Zhang et al., 2017). Roots may select for specific microbial communities by releasing root exudates into the rhizosphere (Edwards et al., 2015; Zhang et al., 2017; Ceja-Navarro et al., 2021). The change in protist diversity might have arisen from the subsequent enrichment and depletion of some protists going from the bulk soil to soybean rhizosphere. Combined with our previous studies, these results suggested that the rhizosphere of soybean had a selective effect on protists similar to the effect on bacteria and fungi (Zhang et al., 2018a,b).

In our study, a greater number of OTUs were depleted rather than enriched in the soybean rhizosphere, suggesting that this compartment has a greater effect on exclusion rather than enrichment of protists in soybean fields. Interestingly, the phagotrophs showed a higher relative abundance in the soybean rhizosphere than in the bulk soil, although more OTUs of phagotrophs were depleted in the rhizosphere. Beyond having niches that fit better to the rhizosphere’s environment (Edwards et al., 2015), the higher density of bacteria in the rhizosphere could provide phagotrophs with more food (Paterson et al., 2007; Shi et al., 2015). Rhizaria was most abundant in both the bulk soil and rhizosphere. Within Rhizaria, Cercomonadida are classified as phagotrophs and are known to be widespread in soils. One study showed that Cercomonadida has several plant rhizosphere colonizers that are more enriched in the rhizosphere than in bulk soil (Turner et al., 2013).

Moreover, some specific protists may become depleted in the rhizospheric environment due to interspecific competition. Apusozoa, Archaeplastida, Hacrobia, and Stramenopiles were all less abundant in the soybean rhizosphere than in the bulk soil compartment, and many OTUs belonging to them were classified into functional groups of phototrophs and saprotrophs. The bulk soil samples were collected from 0 to 20 cm, which partly receives light, while the rhizosphere soil, where the light penetration was minimal. Thus, the rhizosphere is not a favorable place for most of the phototrophs, which is why they are richer in relative abundance in the bulk soil. On the other hand, some phagotrophs, as top predators, not only feed on bacteria but may even feed on other protists (Geisen and Bonkowski, 2017). Presumably, the enrichment of these predators may partly lead to some protists being lost or excluded from the soybean rhizosphere community.

### Various Factors Driving the Composition of Protist Communities in Soybean Fields

We found that spatial location did the most to explain shifts in the composition of protist communities of soybean fields, highlighting the key contribution of historical processes in structuring protist communities of soybean fields (Beck et al., 2015). The belowground compartment was the second-most important factor for explaining the assembly of protist communities. The relative contributions made by location and compartment in structuring microbial communities often differ among studies depending on their sampling scale (Peiffer et al., 2013; Edwards et al., 2015; Zarraonaindia et al., 2015; Santos-Medellín et al., 2017). Since our samples were collected from different ecological regions across China, this corresponds to a large spatial scale for our reported results. In addition, soybean cultivar had a negligible impact on shifts in the composition of rhizosphere protist communities. Previous studies of bacterial communities of farmed rice and maize also identified cultivar as the least explanatory variable among other considered variables (Peiffer et al., 2013; Edwards et al., 2015).

Furthermore, we investigated the influence of climatic and edaphic factors on the protist community. Among these, MAP was clearly the most important acting on protist communities in the bulk soil and soybean rhizosphere. This result contrasts with most prior studies in which soil pH was the main environmental driver of protist community assembly in different soil types of various ecosystems (Bates et al., 2013; Ramirez et al., 2014; Shen et al., 2016). Even among the edaphic factors, calcium showed a stronger impact on protist community assembly than soil pH. This latter pattern was also observed in several studies that investigated protist communities of mosses (Jassey et al., 2014; Heger et al., 2018). However, the detailed mechanism by which calcium influences protist community composition is poorly understood (Heger et al., 2018).

### Less Complex Protist Co-occurrence Networks in the Rhizosphere of Soybean

Non-random protist networks were explored for the two compartments. The rhizosphere clearly harbored a smaller and less complex network structure than did the bulk soil, thus indicating a smaller potential of niche overlap and interactions in the rhizosphere due to the increased resources of the rhizosphere (Zhang et al., 2018c). Enriched resources decrease the frequency of microbial interactions and allow more microorganisms to maintain free-living patterns (Fan et al., 2018). In addition, enriched resources lead a higher proportion of positive correlations in the soybean rhizospheric protist network than in the bulk soil, indicating a higher proportion of cooperative or syntrophic interactions among protists in the rhizosphere assemblages (Fan et al., 2018). We speculated that enriched rhizosphere resources decreased the proportion of competitive relationships.

Modularity refers to highly connected microbes that cluster into a group (Ling et al., 2016). We obtained four and three modules in networks of the bulk soil and soybean rhizospheric soil, respectively. Rhizaria dominated most of the modules of networks of the bulk soil and rhizosphere, indicating a crucial ecological role in soybean fields. Other environmental sampling also showed that Rhizaria lineages are widespread and ubiquitous and play a variety of ecological functions, such as affecting soil moisture, soil bulk density and C microbial biomass (Dumack et al., 2020). The correlations with environmental factors were different among modules. Our results suggested that environmental factors change protist co-occurrence relationships by influencing specific functional modules. The different environmental correlations among modules also imply the role of habitat heterogeneity in module formation (Zhang et al., 2018c).

### The Relationships Between Protists and Other Types of Microorganisms

Soil protists are commonly considered solely bacterivorous (Bonkowski and Brandt, 2002; Koller et al., 2013; Flues et al., 2017). However, some protist taxa will facultatively feed on diverse fungi (Geisen et al., 2016). To better understand how protists are an integral part of this ecological network, we constructed co-occurrence networks that included bacteria, fungi, and protists obtained from the bulk soil and soybean rhizosphere.

The correlations were mainly dominated by interactions between the same taxa, especially for the positive correlations. Microbes belonging to the same taxa are more closely related and typically have similar niches or interactions (Ju and Zhang, 2015). However, we also observed that protists in the rhizosphere mainly interact with bacteria and fungi. The proportion of positive edges correlated with the protists decreased, while the proportion of negative edges correlated with the protists increased to varying degrees. These results might suggest that a higher proportion of predator-prey interactions occur in the rhizosphere. A recent study showed that protists in the rhizosphere might protect plants by feeding on other microorganisms and shifting the taxonomic and functional composition of microorganisms of the rhizosphere (Xiong et al., 2020). Our study demonstrates that rhizospheric protists also have the potential to influence bacterial and fungal communities to influence the rhizospheric co-occurrence relationships of soybean plants. We speculated that protists could form the rhizosphere microbiome by predator-prey interactions to influence the status of soybean plants. Some protist OTUs were identified as keystone species despite protist nodes with the lowest counts in the overall networks. These protist nodes linked a range of bacteria and fungi. This result suggests that protists play a crucial role as an integral part of the microbial co-occurrence network of soybean fields.

## Conclusion

Our work provides detailed insights into differences in the community structure of protists between the bulk soil and soybean rhizosphere and expands our understanding of how location, compartment, and cultivar determine rhizospheric protist communities in soybean fields. Furthermore, as our study explored protist co-occurrence networks and overall network-integrated bacteria, fungi, and protists in rhizosphere and bulk soil, our understanding of interactions in these microbial communities has taken a step forward. Taken together, protists occupy an integral part of microbial networks of soybean fields.

## Data Availability Statement

The datasets presented in this study can be found in online repositories. The names of the repository/repositories and accession number(s) can be found below: https://www.ncbi.nlm.nih.gov/, PRJNA478202.

## Author Contributions

PS, GW, and JZ conceived and designed the review. JZ, PX, and MN wrote the manuscript. All authors contributed to the article and approved the submitted version.

## Conflict of Interest

The authors declare that the research was conducted in the absence of any commercial or financial relationships that could be construed as a potential conflict of interest.

## Publisher’s Note

All claims expressed in this article are solely those of the authors and do not necessarily represent those of their affiliated organizations, or those of the publisher, the editors and the reviewers. Any product that may be evaluated in this article, or claim that may be made by its manufacturer, is not guaranteed or endorsed by the publisher.

## Footnotes

1
http://data.cma.cn/
