# Supplementary material for: Taxonomic Compositions and Co-occurrence Relationships of Protists in Bulk Soil and Rhizosphere of Soybean Fields in Different Regions of China
Source: Front Microbiol. 2021 Sep 17;12:738129. doi: 10.3389/fmicb.2021.738129 (PMC8485050; doi:10.3389/fmicb.2021.738129)
Supplement: Supplementary file 1 [file Data_Sheet_1.docx]

**SUPPLEMENTARY INFORMATION**

**Taxonomic compositions and co-occurrence relationships of protists in bulk soil and rhizosphere of soybean fields at different regions of china**

Jun Zhang, Pengcheng Xing, Mengyu Niu, Gehong Wei, Peng Shi*
*State Key Laboratory of Crop Stress Biology in Arid Areas, Shaanxi Key Laboratory of Agricultural and Environmental Microbiology, College of Life Sciences, Northwest A&F University, Yangling, Shaanxi 712100, PR China*

*Corresponding author.

Postal address: *State Key Laboratory of Crop Stress Biology in Arid Areas, Shaanxi Key Laboratory of Agricultural and Environmental Microbiology, College of Life Sciences, Northwest A&F University, 3 Taicheng Road, Yangling, Shaanxi 712100, PR China.*

Tel.: +86 29 87091175.
*E-mail address*: shipeng27@nwafu.edu.cn (P. Shi)

**Table S1** Sampling sites and environmental variables (soil properties and climatic data) of the 6 sampling sites in soybean fields.

**Table S2**

(A) PERMANOVA testing the effects of location and compartment, and their interaction on beta-diversity.

|  | Df | SS | F | R^2^ | P |
| --- | --- | --- | --- | --- | --- |
| **Bray-Curtis distance** |  |  |  |  |  |
| Location | 5 | 5.6566 | 9.5475 | 0.36638 | 0.0001 |
| Compartment | 1 | 1.2580 | 10.6167 | 0.08148 | 0.0001 |
| Location: Compartment | 5 | 1.4149 | 2.3881 | 0.09164 | 0.0001 |
| Residual | 60 | 7.1097 |  | 0.4605 |  |
| Total | 71 | 15.4392 |  | 1 |  |
| **Weighted UniFrac distance** |  |  |  |  |  |
| Location | 5 | 3.5916 | 12.8864 | 0.42446 | 0.0001 |
| Compartment | 1 | 0.7968 | 14.2934 | 0.09416 | 0.0001 |
| Location: Compartment | 5 | 0.7287 | 2.6144 | 0.08612 | 0.0001 |
| Residual | 60 | 3.3446 |  | 0.39526 |  |
| Total | 71 | 8.4617 |  | 1 |  |

(B) PERMANOVA testing the effects of location, cultivar and their interaction on beta-diversity. Bulk soils were excluded before running this analysis because they lack an assigned soybean cultivar.

|  | Df | SS | F | R^2^ | P |
| --- | --- | --- | --- | --- | --- |
| **Bray-Curtis distance** |  |  |  |  |  |
| Location | 5 | 5.1170 | 8.4407 | 0.44746 | 0.0001 |
| Cultivar | 2 | 0.3288 | 1.3560 | 0.02875 | 0.0812 |
| Location: Cultivar | 10 | 1.6249 | 1.3402 | 0.09164 | 0.0089 |
| Residual | 36 | 4.3648 |  | 0.4605 |  |
| Total | 53 | 11.4355 |  | 1 |  |
| **Weighted UniFrac distance** |  |  |  |  |  |
| Location | 5 | 3.2047 | 11.7156 | 0.51080 | 0.0001 |
| Cultivar | 2 | 0.1499 | 1.3698 | 0.02389 | 0.1286 |
| Location: Cultivar | 10 | 0.9499 | 1.7362 | 0.15140 | 0.0004 |
| Residual | 36 | 1.9695 |  | 0.31392 |  |
| Total | 53 | 6.2739 |  | 1 |  |

**Table S3** Mantel test used to investigate the edaphic and climatic factors correlated with the protist communities in the bulk soil and rhizosphere.

|  | Bulk soil | | Rhizosphere | |
| --- | --- | --- | --- | --- |
|  | R | P | R | P |
| pH | 0.4155 | < 0.001*** | 0.4685 | < 0.001*** |
| TN | 0.2015 | 0.0377* |  |  |
| Ca | 0.5919 | < 0.001*** | 0.5948 | < 0.001*** |
| MAP | 0.6519 | < 0.001*** | 0.6562 | < 0.001*** |
| AI |  |  | 0.6512 | < 0.001*** |

A forward selection procedure determined the best explanatory variables. TN, total nitrogen; pH, soil pH; MAP, mean annual precipitation; AI, aridity index.

**Table S4**

1. Mantel analyses to link Euclidean distance of network modular eigengenes of the bulk soil to Euclidean distances of standardized soil physicochemical properties and climatic data, respectively.

|  | Module I | |  | Module II | |  | Module III | |  | Module IV | |
| --- | --- | --- | --- | --- | --- | --- | --- | --- | --- | --- | --- |
|  | *r* | *p* |  | *r* | *p* |  | *r* | *p* |  | *r* | *p* |
| *Soil physicochemical properties* | | | | | | | | | | | |
| pH | -0.05 | 0.733 |  | **0.19** | **0.031** |  | **0.24** | **0.020** |  | 0.11 | 0.094 |
| OC | 0.01 | 0.410 |  | 0.11 | 0.093 |  | **0.56** | **0.001** |  | **0.16** | **0.043** |
| TN | 0.06 | 0.196 |  | 0.11 | 0.081 |  | **0.15** | **0.045** |  | **0.28** | **0.006** |
| CN | 0.05 | 0.200 |  | 0.10 | 0.112 |  | **0.16** | **0.041** |  | **0.27** | **0.016** |
| AN | -0.04 | 0.714 |  | 0.08 | 0.129 |  | **0.16** | **0.037** |  | -0.06 | 0.772 |
| AP | -0.05 | 0.761 |  | **0.19** | **0.021** |  | 0.12 | 0.073 |  | 0.01 | 0.376 |
| AK | -0.01 | 0.461 |  | 0.01 | 0.416 |  | 0.12 | 0.076 |  | 0.17 | 0.051 |
| Ca | 0.02 | 0.297 |  | 0.12 | 0.087 |  | **0.48** | **0.001** |  | **0.32** | **0.004** |
| Mg | 0.05 | 0.232 |  | 0.09 | 0.132 |  | **0.40** | **0.001** |  | **0.42** | **0.001** |
| Clay | 0.01 | 0.365 |  | 0.08 | 0.135 |  | **0.16** | **0.042** |  | **0.18** | **0.041** |
| Silt | 0.07 | 0.152 |  | 0.04 | 0.246 |  | **0.21** | **0.024** |  | **0.21** | **0.031** |
| Sand | 0.05 | 0.200 |  | 0.05 | 0.220 |  | **0.17** | **0.045** |  | **0.26** | **0.013** |
| *Climatic data* | | | | | | | | | | | |
| MAT | 0.06 | 0.175 |  | 0.13 | 0.057 |  | **0.46** | **0.001** |  | **0.54** | **0.001** |
| MAP | 0.01 | 0.333 |  | 0.09 | 0.131 |  | **0.55** | **0.001** |  | **0.44** | **0.001** |
| RH | 0.03 | 0.334 |  | 0.01 | 0.422 |  | **0.28** | **0.004** |  | **0.46** | **0.001** |
| PE | 0.01 | 0.408 |  | 0.08 | 0.139 |  | **0.24** | **0.001** |  | **0.51** | **0.001** |
| AI | 0.03 | 0.286 |  | 0.08 | 0.163 |  | 0.53 | 0.003 |  | **0.45** | **0.001** |

pH, soil pH; OC, organic carbon; TN, total nitrogen; CN, the ratio of microbial biomass carbon and nitrogen; AN, available nitrogen; AP, available phosphorous; AK, available potassium; MAT, mean annual temperature; MAP, mean annual precipitation; RH, relative humidity; PE, potential evapotranspiration AI, aridity index.

1. Mantel analyses to link Euclidean distance of network modular eigengenes of the rhizosphere to Euclidean distances of standardized soil physicochemical properties and climatic data, respectively.

|  | Module I | |  | Module II | | |  | Module III | |  |
| --- | --- | --- | --- | --- | --- | --- | --- | --- | --- | --- |
|  | *r* | *p* |  | *r* | *p* | |  | *r* | *p* |  |
| *Soil physicochemical properties* | | | | | |  |  |  |  |  |
| pH | 0.02 | 0.196 |  | -0.11 | 0.997 | |  | -0.08 | 0.980 |  |
| OC | -0.02 | 0.735 |  | -0.01 | 0.608 | |  | 0.01 | 0.459 |  |
| TN | **0.08** | **0.041** |  | 0.04 | 0.215 | |  | -0.16 | 0.990 |  |
| CN | **0.32** | **0.001** |  | -0.16 | 0.997 | |  | -0.08 | 0.969 |  |
| AN | **0.15** | **0.004** |  | -0.12 | 0.986 | |  | -0.01 | 0.626 |  |
| AP | -0.02 | 0.97 |  | -0.09 | 0.959 | |  | -0.17 | 0.990 |  |
| AK | **0.23** | **0.001** |  | 0.01 | 0.499 | |  | -0.08 | 0.959 |  |
| Ca | **0.17** | **0.001** |  | -0.09 | 0.985 | |  | -0.04 | 0.890 |  |
| Mg | 0.05 | 0.101 |  | 0.06 | 0.047 | |  | -0.01 | 0.629 |  |
| Clay | **0.36** | **0.001** |  | -0.20 | 0.975 | |  | -0.13 | 0.998 |  |
| Silt | **0.28** | **0.001** |  | -0.10 | 0.989 | |  | -0.13 | 0.999 |  |
| Sand | **0.31** | **0.001** |  | -0.13 | 0.987 | |  | -0.16 | 0.999 |  |
| *Climatic data* | | | | | |  |  |  |  |  |
| MAT | **0.15** | **0.001** |  | **0.07** | **0.024** | |  | -0.11 | 0.997 |  |
| MAP | 0.03 | 0.131 |  | -0.02 | 0.704 | |  | -0.13 | 0.997 |  |
| RH | **0.21** | **0.001** |  | -0.08 | 0.911 | |  | -0.16 | 0.999 |  |
| PE | 0.03 | 0.292 |  | 0.06 | 0.127 | |  | -0.18 | 0.999 |  |
| AI | **0.11** | **0.003** |  | -0.04 | 0.920 | |  | -0.13 | 0.999 |  |

pH, soil pH; OC, organic carbon; TN, total nitrogen; CN, the ratio of microbial biomass carbon and nitrogen; AN, available nitrogen; AP, available phosphorous; AK, available potassium; MAT, mean annual temperature; MAP, mean annual precipitation; RH, relative humidity; PE, potential evapotranspiration AI, aridity index.

**Table S5** Topological properties of the total microbial co-occurrence networks in bulk soil and rhizosphere of the soybean fields in China and their associated random networks.

| Parameters | Compartment | |
| --- | --- | --- |
|  | Bulk soil | Rhizosphere |
| **Empirical networks** |  |  |
| Number of nodes | 601 | 392 |
| Number of edges | 1499 | 1093 |
| Number of positive correlations | 1245（83.06%） | 1045（95.61%） |
| Number of negative correlations | 254（16.94%） | 48（4.39%） |
| Average degree | 4.988 | 5.577 |
| APL | 5.692 | 5.977 |
| ACC | 0.439 | 0.477 |
| Modularity | 0.751 | 0.734 |
| **Random networks** |  |  |
| APL | 4.147 ± 0.016 | 3.656 ± 0.013 |
| ACC | 0.008 ± 0.002 | 0.014 ± 0.003 |
| Modularity | 0.439 ± 0.005 | 0.403 ± 0.006 |

**
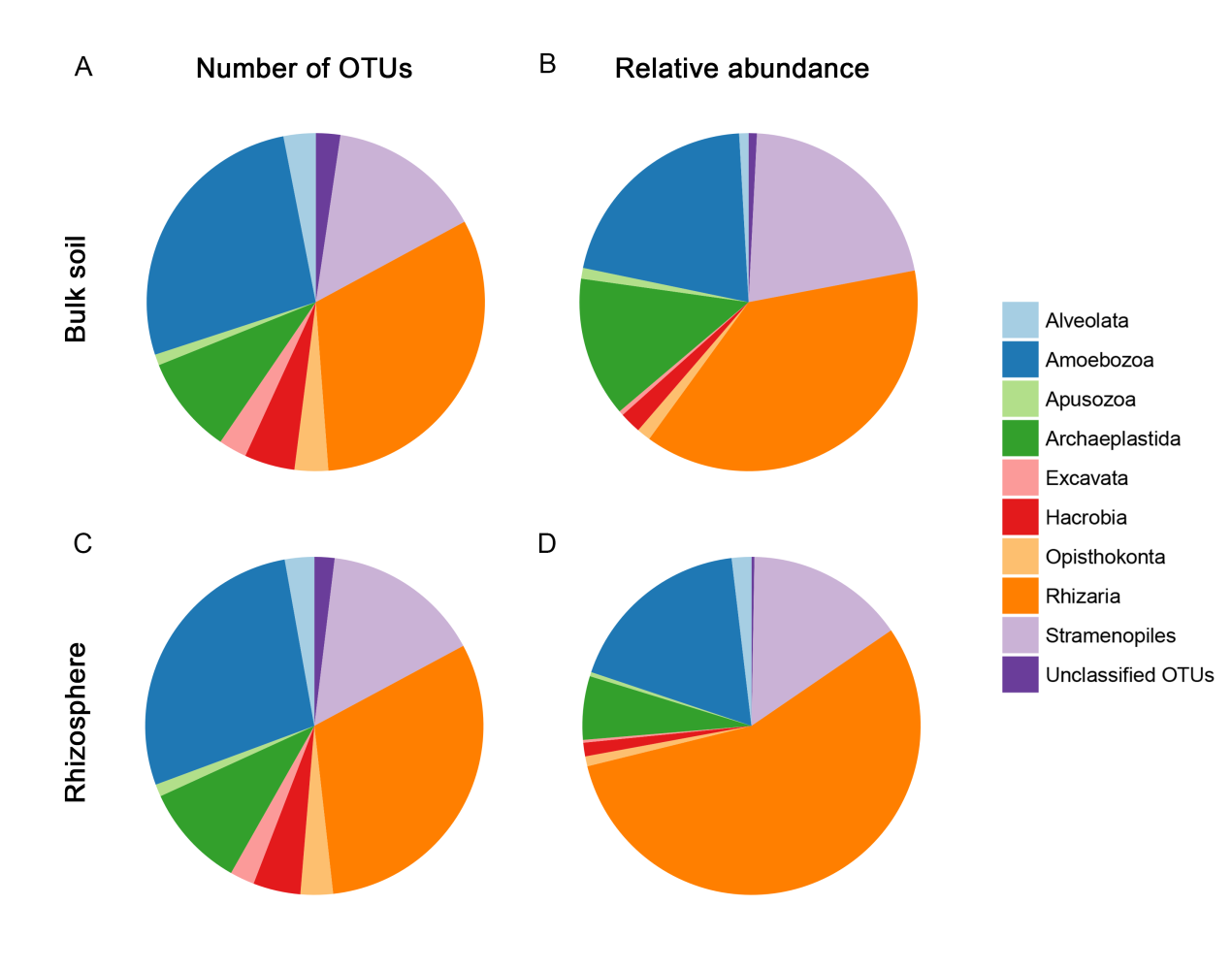
**

**Fig. S1.** Taxonomic compositions of the protist community retrieved from the bulk soil (A and C) and rhizosphere (B and D). Pie charts display the relative abundance and the number of OTUs of the protists at a high level of taxonomic assignment.

**
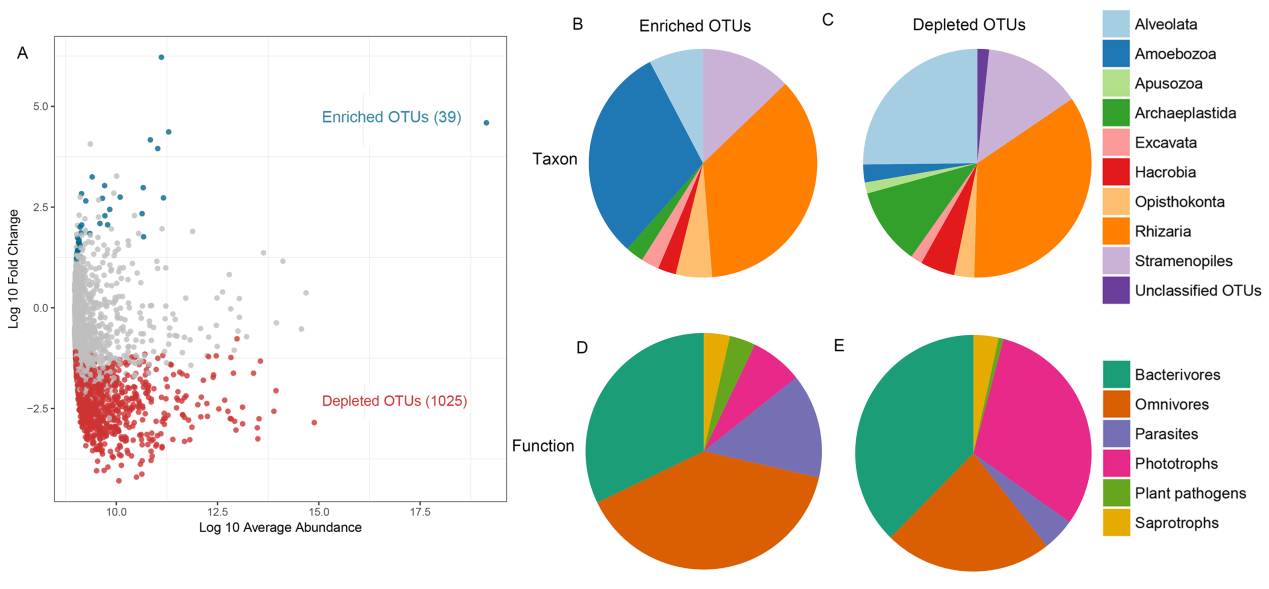
**

**Fig. S2.** Enrichment and depletion of OTUs, going from the bulk soil to rhizosphere of soybean, as calculated by differential abundance analysis. (A) Each point represents an individual OTU, blue and red points represent OTUs which had a significantly higher and lower relative abundance in the rhizosphere, respectively. Taxonomic (B and C) and functional (D and E) compositions of the protist community that were enriched or depleted in the soybean rhizosphere.


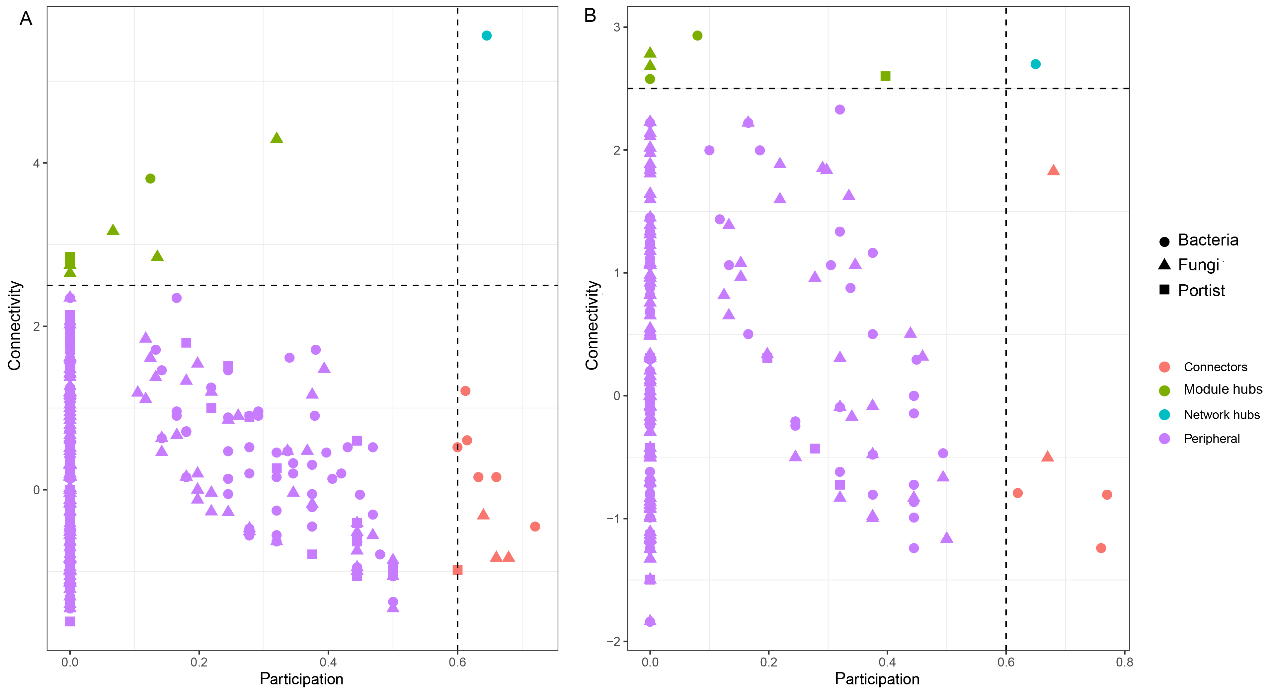


**Fig. S3.** Network roles of analysing module feature at OTU level for total microbial networks in bulk soil (A) and rhizosphere (B) of the soybean fields.
